# Hiccups: An Atypical Manifestation of Miliary Tuberculosis and Myocardial Ischemia

**DOI:** 10.7759/cureus.53595

**Published:** 2024-02-05

**Authors:** Amit Ramrattan, Carina Aguilar

**Affiliations:** 1 Internal Medicine, Port of Spain General Hospital, Port of Spain, TTO; 2 Radiology, Port of Spain General Hospital, Port of Spain, TTO

**Keywords:** mechanism and pathway for hiccups, elderly patients, hiv aids, non-st segment elevation myocardial infarction (nstemi), miliary tuberulosis, persistent hiccups

## Abstract

Hiccups are a benign, self-limiting condition caused by intermittent spasmodic contractions of the diaphragm. However, its persistence may be indicative of a more ominous underlying condition, and its manifestation is seen in myocardial ischemia/infarction and tuberculosis (TB). We present two unique cases where persistent hiccups lead to the diagnosis of miliary tuberculosis in one patient and myocardial infarction in the other. It stresses the importance that such a benign presentation may be a warning for a more sinister pathology and thus requires extensive work-up in high-risk and senior individuals.

## Introduction

Brief episodes of hiccups are a nuisance, yet a common part of life. The symptoms can be so debilitating that patients present to emergency departments for relief. Hiccups are commonly associated with gastric distension, typically from overeating, carbonated beverages, aerophagia, post-upper endoscopies, or excessive alcohol consumption [1]. It can be defined as transient, persistent, chronic, or intractable if it lasts less than one day, more than 48 hours, or more than one month, respectively [2]. Persistent hiccups can indicate a serious pathology. Though pneumonia is a known cause of hiccups, few case descriptions exist. Furthermore, hiccups as a manifestation of tuberculosis (TB) are exceedingly rare [3]. The first description of cardiovascular hiccups was dated back in 1939 [4] but only came to the forefront in 1993 by Launois et al. [5]. Acute myocardial infarctions (AMI) were observed to be the most common cause of cardiovascular hiccups. This paper highlights two cases at the Port of Spain General Hospital in Port of Spain, Trinidad and Tobago, where the presentation of hiccups led to investigations and the portentous findings of military TB and AMI.

## Case presentation

Case one

A 62-year-old male presented to Port of Spain General Hospital with hiccups and shortness of breath for two weeks. There was no medical history or hospitalization, but he had a history of incarceration. He had a positive history of intermittent fever, drenching night sweats, and weight loss that was unquantifiable, and he was a chronic smoker with a smoking history of 30 pack years. He also had a non-productive cough. Physical examination revealed a blood pressure (BP) of 121/72 mmHg, a pulse rate (PR) of 127 beats per minute, tachypnea at a respiratory rate (RR) of 25 breaths per minute, pulse oximetry on room air at 95%, and a temperature of 36.6°C. Auscultation revealed normal cardiac sounds and diminished breath sounds in the lower zones, with no crepitations and scattered rhonchi. The patient had oral candidiasis and no palpable cervical or axillary lymph nodes, and the rest of the abdominal examination yielded no abnormalities.

Laboratory investigations of the patient are tabulated in Table 1.

**Table 1:** Blood investigation results of patient one pH: acid-base balance of the blood: pCO_2_: partial pressure of carbon dioxide; pO_2_: partial pressure of oxygen; HCO3: bicarbonate

Test	Result	Normal range
Hemoglobin	11.5 g/dl	13-17 g/L
Mean corpuscular volume	91.1 fL	83-101 fL
White blood cell count	5 x10^3^ /µL	4-10 x10^3^ /µL
Platelet count	196 x10^3^ /µL	150-410 x10^3^ /µL
Sodium	128 mmol/L	136-145 mmol/L
Potassium	5.6 mmol/L	3.5-5.1 mmol/L
Chlorine	93.5 mmol/L	98.0-107.0mmol/L
Blood urea nitrogen	16.1 mg/dl	6.0-23.0 mg/dl
Creatinine	0.8 mg/dl	0.7-1.2 mg/dl
D-dimer	2176 ng/ml	Less than 250 ng/ml
pH	7.47	7.35- 7.45
pCO_2_	28 mmHg	35-45 mmHg
pO_2_	78 mmHg	75-100 mmHg
HCO3	21.3 mEq/L	22-26 mEq/L

The liver function test was normal, apart from a low albumin level of 2.9 g/dl (normal range: 3.5-5.1 g/dl). The electrocardiogram (ECG) showed sinus tachycardia but no other abnormalities.

**Figure 1:**
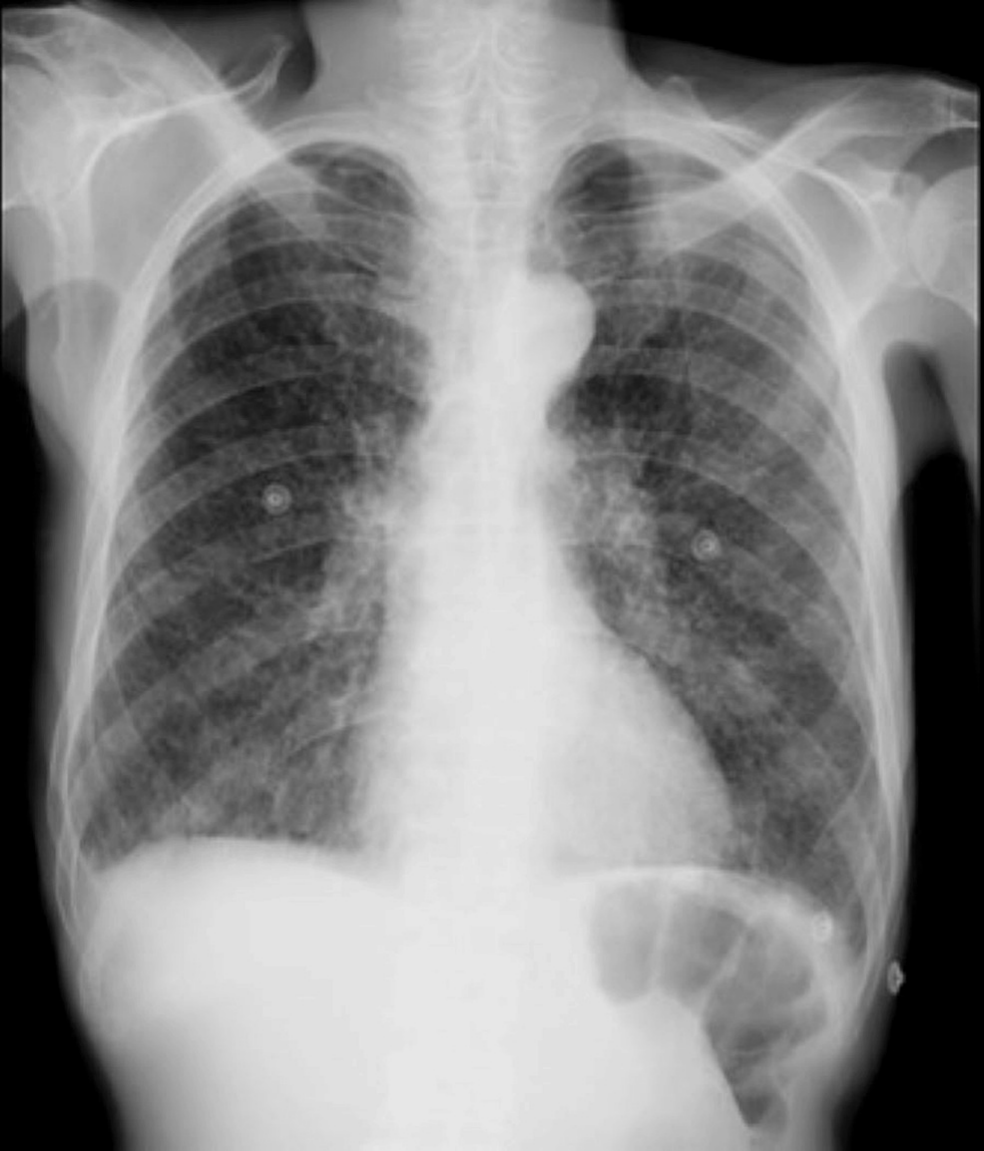
Chest X-ray of patient one The image shows bilateral, innumerable, diffusely scattered 1 mm –4 mm opacities that were suggestive of an infective etiology and suspicious for miliary pulmonary nodules.

A chest X-ray (CXR) was performed (Figure 1).

**Figure 2:**
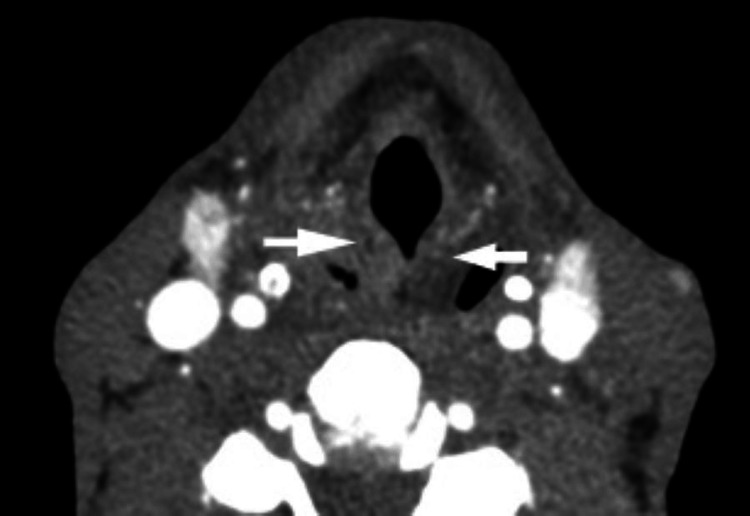
Neck CT scan of patient one The image shows bilateral symmetrical soft tissue thickening of the aryepiglottic folds (white arrows), up to 1.0 cm in diameter, without any focal enhancement mass being noted, and no enlarged cervical nodes were present.

A human immunodeficiency virus (HIV) test was subsequently done, which was positive. A computer tomography (CT) of the neck was done (Figure 2).

**Figure 3:**
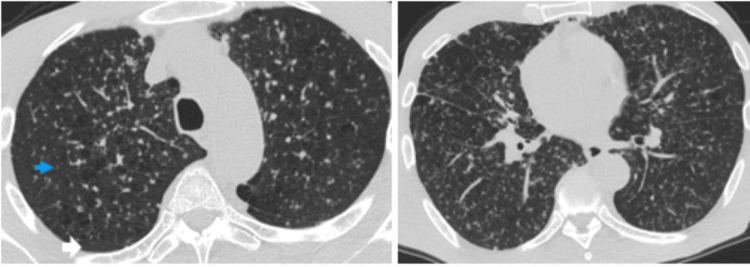
Chest CT of patient one The image shows miliary pulmonary nodules (blue arrowhead). There was no evidence of pulmonary masses, but the background lung shows emphysema with few bilateral upper lobe centrilobular bullae in and small subpleural bullae in the left apex (white arrow). No enlarged mediastinal or hilar nodes or fibrocavitatory changes were present.

A CT scan of the chest was also done (Figure 3). 

The patient was placed in an isolation room with protocols for barrier nursing. Considering the non-productive cough, a saline nebulizer was given to induce sputum. Acid-fast bacilli testing was negative by three early morning samples. Sputum for the TB polymerase chain reaction (PCR) did not detect *Mycobacterium tuberculosis*. The COVID-19 PCR and Influenza A and B PCR tests were also negative. An infectious disease consult was requested, and the patient was managed with co-trimoxazole 1,440 mg per oral (PO) every eight hours, enoxaparin 40 mg subcutaneous (SC) once daily (OD) for thromboembolism prophylaxis, and omeprazole 20 mg OD. The CD4 count and viral load samples were taken for testing, and the patient was subsequently transferred to Caura Hospital for further management of miliary TB.

Case two

An 84-year-old male with known hypertensive heart disease and cerebrovascular disease presented to Port of Spain General Hospital with left-sided weakness and vomiting for one day. His BP on admission was 159/89 mmHg, PR was 75 beats per minute, RR was 16 breaths per minute, pulse oximetry was 99% room air, temperature was 36.3°C, and a random blood sugar was 111 mg/dl. A physical examination yielded normal cardiac sounds, normal vesicular breath sounds, and a normal abdomen. The patient’s Glasgow coma scale (GCS) was 15/15, but he did have hemiparesis on the right with 3/5 power. Blood investigations were done on the patient (Table 2).

**Table 2:** Blood investigation results of patient two

Test	Result	Normal range
Hemoglobin	14 g/dl	13-17 g/L
Mean corpuscular volume	86.9 fL	83-101 fL
White blood cell count	7.7 x10^3^ /µL	4-10 x10^3^ /µL
Platelet count	177 x10^3^ /µL	150-410 x10^3^ /µL
Sodium	139 mmol/L	136-145 mmol/L
Potassium	3.0 mmol/L	3.5-5.1 mmol/L
Chlorine	98.8 mmol/L	98.0-107.0mmol/L
Blood urea nitrogen	10.7 mg/dl	6.0-23.0 mg/dl
Creatinine	0.9 mg/dl	0.7-1.2 mg/dl

**Figure 4:**
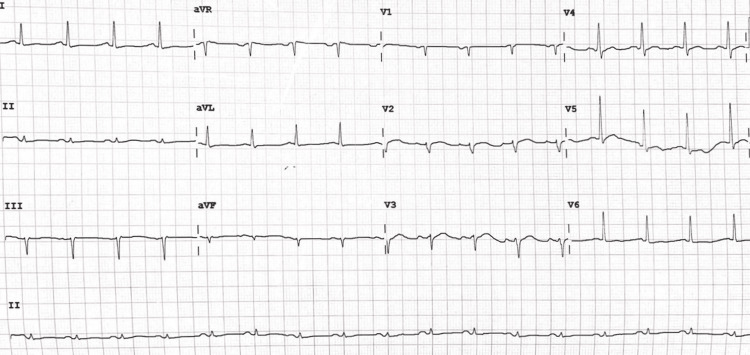
The ECG of patient two taken on admission had had no significant findings

Liver function tests and thyroid function tests were within normal limits. The ECG on admission (Figure 4) was normal, and a high-sensitive troponin was within normal limits.

**Figure 5:**
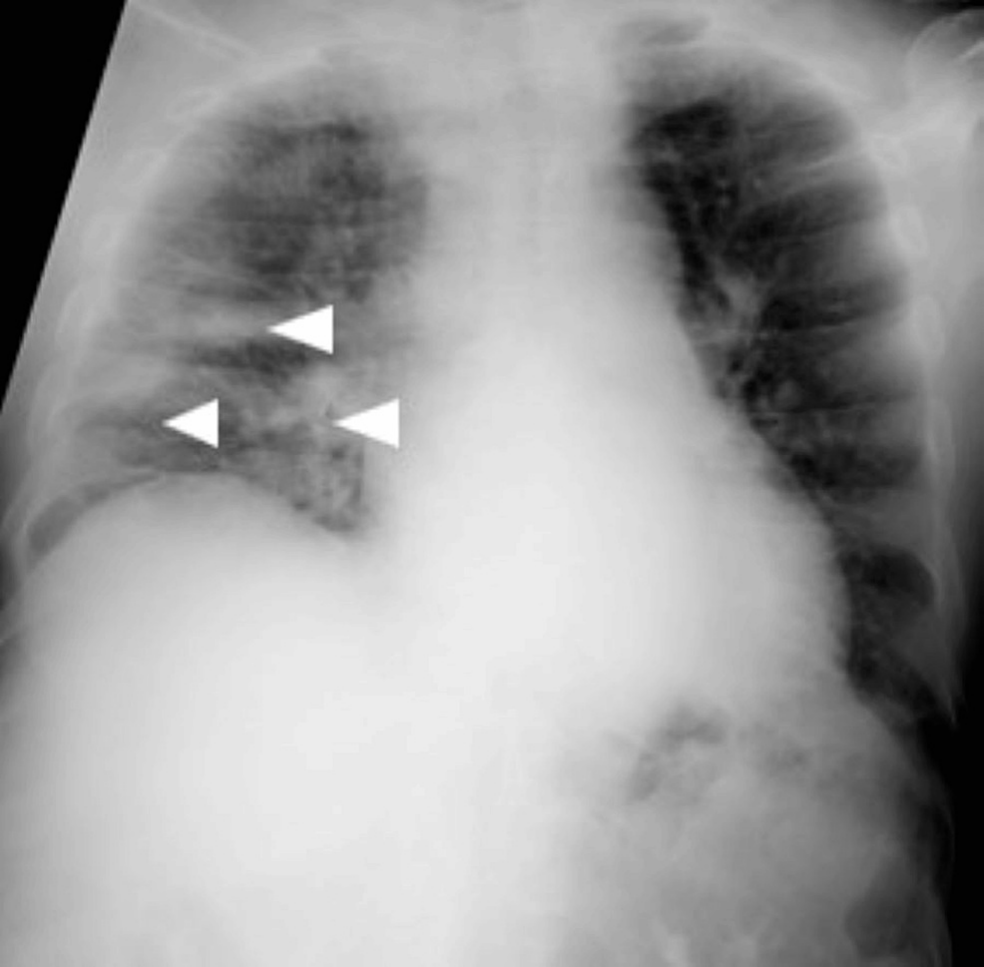
Chest X-ray of patient two The image revealed a right lung volume reduction with patchy and nodular air space opacities in the mid-to-lower zones (arrowheads) and borderline cardiomegaly with a cardiothoracic ratio of 0.55.

A CXR was also performed (Figure 5).

**Figure 6:**
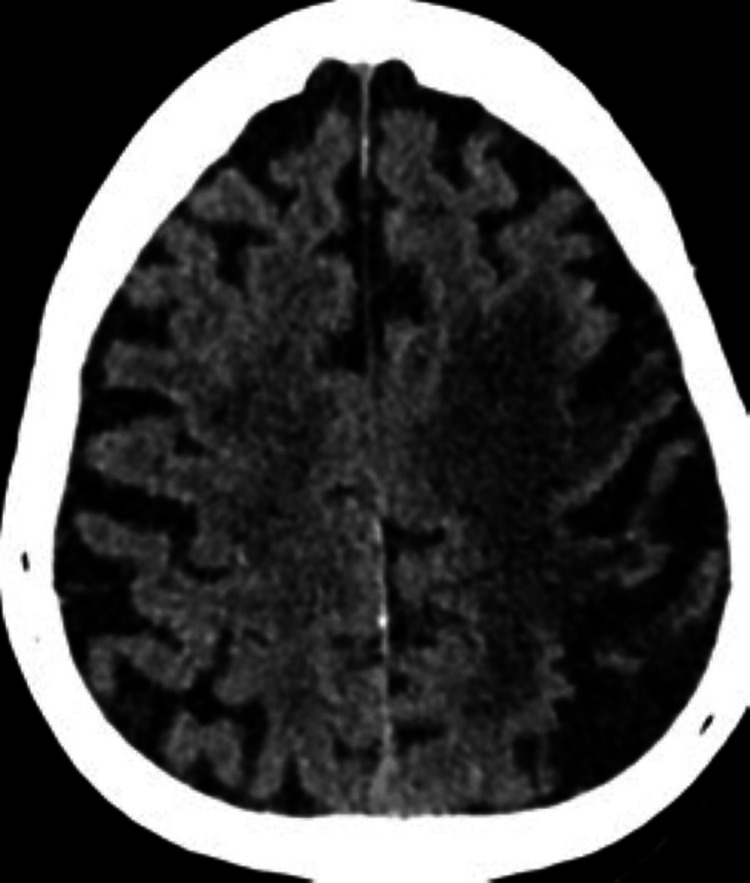
Brain CT of patient two The image shows chronic infarcts in the left superior frontal and parietal lobes with encephalomalacia.

**Figure 7:**
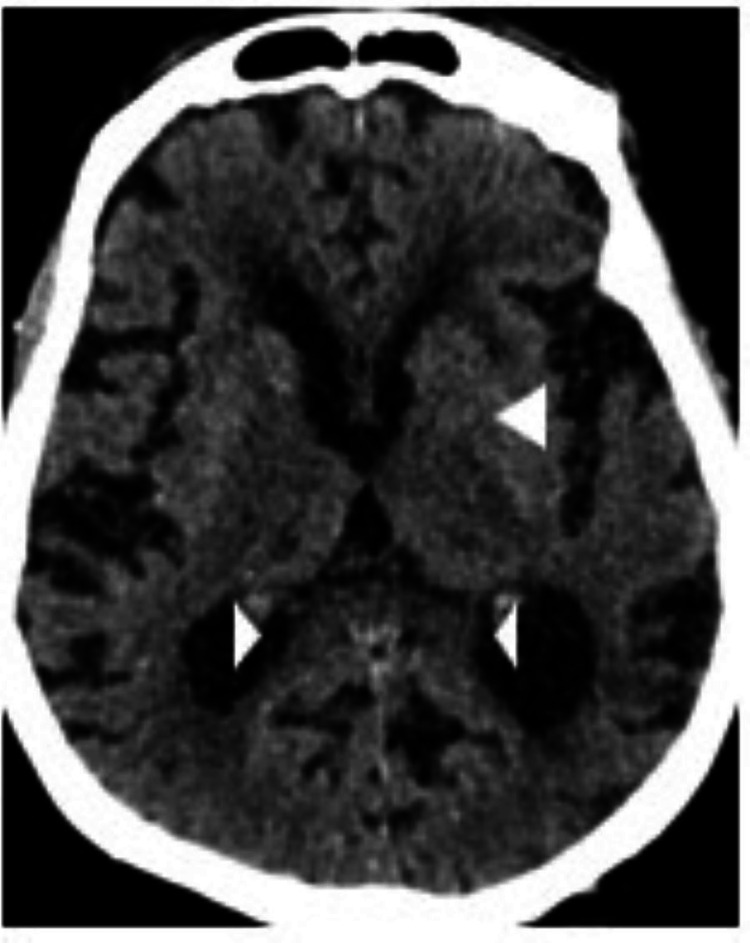
Brain CT of patient two The image shows bilateral thalamic and left external capsule chronic infarcts (top arrowhead) and age-related involutional changes noted with the widening of the sulci and dilatation of the ventricles (bottom arrowheads). There are periventricular hypodensities in keeping with chronic small vessel ischemic changes.

A CT scan of the brain was performed in light of the neurological signs (Figures 6, 7).

**Figure 8:**
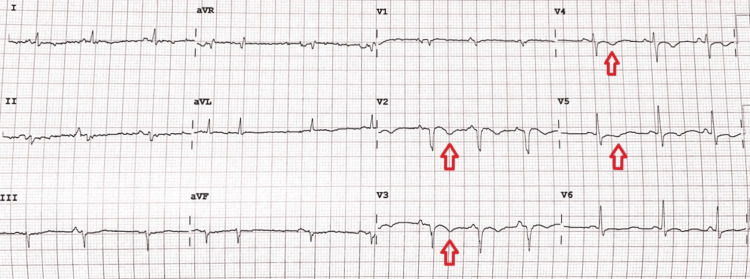
Repeat ECG performed for patient two The image shows ECG changes of new anterior and lateral wall T-wave inversions (red arrows).

The patient was treated for an acute ischemic stroke with aspirin 162 mg OD, rosuvastatin 20 mg nocte, and omeprazole 40mg OD. Ceftriaxone 2g IV OD was commenced to treat pneumonia. One day into his hospital stay, he developed hiccups and was given a trial of chlorpromazine 25 mg PO three times daily (TDS). On day two, the patient then developed a hypotensive episode with BP of 88/57 mmHg and PR of 137 beats per minute. At this point, the patient was given boluses of normal saline and was placed on continuous cardiac monitoring. Serial troponins were done, and on stabilizing, a repeat ECG was performed (Figure 8).

The new ECG changes, in addition to a troponin rise from 16.2 to 64.6 pg/ml (normal range: 12.7-24.9 pg/ml), meant the patient had an anterolateral wall non-ST elevation myocardial infarction (NSTEMI). He was then loaded with aspirin 162 mg and clopidogrel 300 mg, which were continued at dosages of 81 mg and 75 mg daily, along with enoxaparin 60 mg SC BD. The patient’s hiccups had resolved by the fourth day of admission. Due to resource limitations, a coronary angiogram could not be done.

## Discussion

The hiccup reflex [6, 7] is an elaborate interconnected system involving three components: the afferent, central, and efferent components. The afferent component comprises signals from the distal esophagus, stomach, and abdominal part of the diaphragm that travel as part of the phrenic nerve, the vagus, and the sympathetic chain, T6-12 chain branches that feed signals to the central component that comprises the medulla, upper spinal cord C3-5, and the reticular formation. Information from this central component then feeds into the efferent component, where the nerves innervate the diaphragm via the phrenic nerve, the external intercostals and scalene muscles via accessory nerves, and the glottic structures and esophagus via the recurrent laryngeal nerve. The most significant muscle group involved is the diaphragm, where the left hemidiaphragm has been found to be affected in most cases [8]. Any process affecting these three components can trigger hiccups.

Hiccups are usually considered benign as they are self-limited with spontaneous resolution. However, if persistent for more than 48 hours, it can be the result of more threatening etiologies [9], which include meningitis, encephalitis, stroke, and central nervous system tumors that affect the central component. Afferent and efferent components can be stimulated to cause hiccups in the presence of mediastinal tumors, gastroesophageal reflux disease, esophageal tumors, or induced by drugs like steroids, anti-parkinsonian drugs, and antipsychotic medications.

The first case presentation highlights a newly diagnosed HIV patient with miliary TB who has persistent hiccups lasting two weeks. The proposed mechanisms include not only phrenic nerve irritation by his underlying lung inflammation but also possible irritation of the recurrent laryngeal nerve. The bilateral thickening of the aryepiglottic folds seen on imaging of the neck suggests a chronic inflammatory picture, as the patient was not in any respiratory distress when seen. There have been a few case descriptions of pneumonia presenting with hiccups [10-13] and TB presenting with hiccups [3, 14-15]. Given the rising incidence of TB globally [16], clinicians must now be vigilant in investigating persistent or intractable hiccups.

The second case presentation highlighted hiccups as an atypical presentation of ACS. The patient’s sudden decompensation on the ward, with subsequent investigations revealing an NSTEMI, meant that underlying myocardial ischemia was triggering the hiccups. Hiccup has been shown to be an atypical symptom of myocardial ischemia. Culic et al. [17] studied its relationship with myocardial infarction regions and found that hiccups accounted for 5.3%, 2.1%, and 1.1% of the inferior, lateral, and anterior wall infarctions, respectively. Similar observational studies by Sinha et al. [18] and Pasceri et al. [19] concluded that hiccups were more common in territories with inferior wall infarction than in other territories. This is likely due to an ischemic insult in this region activating the afferent component of the hiccup reflex, which includes the vagus, phrenic, and sympathetic nerves. The case presentation shows an anterolateral NSTEMI and likely left anterior descending artery disease. Due to resource limitations, though this patient would have benefited from a coronary angiogram and possible angioplasty, this was not available. However, this case highlights that in an elderly patient with hiccups of an uncertain etiology, electrocardiography is necessary to look for a cardiogenic cause.

## Conclusions

Hiccups, though benign, can be indicative of a more serious pathology, especially in the elderly. Acute coronary syndrome, pneumonia, and, by extension, tuberculosis, carry significant morbidity and mortality. It is therefore imperative that patients with persistent hiccups be investigated for atypical causes, as they may lack physical signs and symptoms that would point to a likely cause.
